# Examining the Presence and Determinants of Operational Momentum in Childhood

**DOI:** 10.3389/fpsyg.2013.00325

**Published:** 2013-06-10

**Authors:** André Knops, Steffen Zitzmann, Koleen McCrink

**Affiliations:** ^1^Department of Psychology, Humboldt University at Berlin, Berlin, Germany; ^2^Barnard College, Columbia University, New York, NY, USA

**Keywords:** approximate calculation, non-symbolic calculation, mental number line, development, space and numbers, attention, numerical cognition

## Abstract

The operational momentum (OM) effect describes a systematic bias in estimating the outcomes of simple addition and subtraction problems. Outcomes of addition problems are overestimated while outcomes of subtraction problems are underestimated. The origin of OM remains debated. First, a flawed uncompression of numerical information during the course of mental arithmetic is supposed to cause OM due to linear arithmetic operations on a compressed magnitude code. Second, attentional shifts along the mental number line are thought to cause OM. A third hypothesis explains OM in 9-month olds by a cognitive heuristic of accepting more (less) than the original operand in addition (subtraction) problems. The current study attempts to disentangle these alternatives and systematically examines potential determinants of OM, such as reading fluency which has been found to modulate numerical–spatial associations. A group of 32 6- and 7-year-old children was tested in non-symbolic addition and subtraction problems, in which they had to choose the correct outcome from an array of several possible outcomes. Reading capacity was assessed for half of the children while attentional measures were assessed in the other half. Thirty-two adults were tested with the identical paradigm to validate its potential of revealing OM. Children (and adults) were readily able to solve the problems. We replicated previous findings of OM in the adults group. Using a Bayesian framework we observed an inverse OM effect in children, i.e., larger overestimations for subtraction compared to addition. A significant correlation between children’s level of attentional control and their propensity to exhibit OM was observed. The observed pattern of results, in particular the inverse OM in children is hard to reconcile with the previously proposed theoretical frameworks. The observed link between OM and the attentional system might be interpreted as evidence partially supporting the attentional shift hypothesis.

## Introduction

Along with a variety of species humans possess an untrained and non-symbolic “number sense,” which yields representations of numerical magnitude that can then be used productively in arithmetic operations such as addition and subtraction (Gallistel, 1990; Wynn, 1992; McCrink and Wynn, 2004; Barth et al., 2005; Cordes et al., 2007; Nieder and Dehaene, 2009). When estimating the outcome of simple mental calculation problems, adults systematically overestimated the outcomes of addition problems and underestimated the outcomes of subtraction problems (McCrink et al., 2007). Analogous to a perceptual phenomenon called representational momentum (Freyd and Finke, 1984) – in which adults misperceive the position at which a moving object disappears in the direction of the movement – this effect was termed operational momentum (OM). OM was observed for both symbolic (e.g., Arabic numerals) and non-symbolic notations (e.g., arrays of objects), implying a notation-independent mechanism which uses semantic and abstract magnitudes as input (Knops et al., 2009b). OM is also observed in paradigms using different response modalities (i.e., choosing from a number of responses, or pointing to the estimated outcome on a linear number scale), suggesting that its origin lies at a central cognitive processing level (Pinhas and Fischer, 2008).

In order to properly detail the existing theoretical hypotheses which attempt to explain OM in approximate calculation, we must first introduce two notions that are crucial for the understanding of the proposed mechanisms. Originally (McCrink et al., 2007; Knops et al., 2009b) the OM bias was explained by mechanisms which describe the underlying numerical magnitude representations as: (a) logarithmically compressed and (b) spatially oriented, with smaller numbers located left from larger numbers. The adult humans tested in these studies possess a cognitive system that enables them to perceive and process numerical magnitude information in an approximate, analog fashion – the aforementioned “number sense,” or approximate number system (ANS). The ANS yields a sense of a given numerical magnitude by activating a fixed position along a numerically ordered continuum, commonly referred to as the Mental Number Line (MNL). Crucially, the MNL is hypothesized to be logarithmically compressed; that is, distances between neighboring numbers decrease logarithmically inversely to the numbers’ magnitudes. Due to the noisiness of activation signals in the ANS, activation at a given position on the MNL will also partially activate adjacent positions. Although still under debate, mounting evidence from behavioral (Moyer and Landauer, 1967; Izard and Dehaene, 2008) and computational studies (Dehaene and Changeux, 1993) suggests that the mental magnitude representation is logarithmically compressed. Most central to the current study, recent data from single-unit recordings has been used to directly test the assumption of a logarithmic compression with fixed variability against alternative scales such as a linear scale with increasing variability (Nieder and Miller, 2003). Models assuming compressed scales yielded better fit indices than a linear scale both during perception of number stimuli and during maintenance in memory. Moreover, compressed scaling of symbolic numbers has been demonstrated to persist in educated Western adults (Viarouge et al., 2010). In a series of experiments adults were asked to judge whether a given sequence of numbers contained too many small numbers or too many large ones. Participants judged as random those sequences that oversampled small numbers. And finally, while scaling of symbolic numbers may linearize over time (Siegler and Opfer, 2003), possibly due to education, non-symbolic numerosities have been found to be mapped in a non-linear way in adults (Dehaene et al., 2008). Nevertheless, some researchers suggest a linearly scaled mental magnitude representation with increasing variability as numerical magnitude increases (Gallistel and Gelman, 2000; Brannon et al., 2001; Ebersbach et al., 2008; Gallistel, 2011; Stoianov and Zorzi, 2012). Indeed, when asking children to place numbers on a spatial scale (e.g., a line) according to their cardinal value the observed mappings change from a logarithmic mapping scheme to a linear mapping scheme as a function of number knowledge and familiarity with numerical concepts (Siegler and Opfer, 2003). Children exhibited a linear mapping scheme for familiar number ranges (e.g., 1–100 for second and fourth graders) and a logarithmic mapping scheme in an unfamiliar number range (e.g., 1–1000 for second and fourth graders) (Siegler and Opfer, 2003; Berteletti et al., 2010). It is unclear to what extent these mapping schemes reflect the scaling schema of the underlying representation, however (Karolis et al., 2011). In sum, we think there is good evidence for a compressed numerical magnitude representation with fixed variability, especially for non-symbolic numerosity information.

Several lines of evidence support the notion of a spatially oriented mental magnitude representation. The classic Spatial–Numerical Association of Response Codes (SNARC) effect implies an association of numerical magnitude representation with external space (Dehaene et al., 1993). In this phenomenon, left-side responses are faster for small numbers, while right-side responses are faster for larger numbers, providing evidence for a spatially oriented MNL in which adults associate small numbers with the left side of space and large numbers with the right. The left-to-right orientation was suggested to result from reading habits in particular societies, such as the French-speaking (left-to-right reading and writing) sample tested by Dehaene et al. (1993); the phenomenon was attenuated in Iranian participants (right-to-left reading and writing) in relation to the number of years they had been in France (Dehaene et al., 1993). Shaki et al. (2009, p. 331) found that the SNARC effect was reversed in Palestinian participants who read both words and numbers from right to left, bolstering the idea that directional reading habits (e.g., left-to-right in Western cultures) “enables the association between numbers and space to become significant,” which in turn may lead to differentially oriented mental number representations depending on cultural and situational variables (Bächtold et al., 1998). The cultural impact on the SNARC effect and the assumed underlying MNL representation is also supported by studies on the developmental trajectory of this phenomenon. The majority of studies investigating the spontaneous spatial orientation of the MNL in children using classic SNARC-like tasks failed to observe significant results before the age of 9 years (Berch et al., 1999; van Galen and Reitsma, 2008; Imbo et al., 2012). However, some recent work using child-friendly paradigms has called this into question, with some evidence for culturally appropriate spatial mapping of small-large magnitudes as early as the preschool years (Opfer et al., 2010; Patro and Haman, 2012). Recent evidence suggests that initial spatial biases become strengthened or weakened depending on the nature of the schooling that children receive (Shaki et al., 2012). Thus, it is conceivable that existing spatial biases consolidate with increasing reading proficiency. Further, semantic activations of magnitudes cause spatial shifts of attention. Fischer et al. (2003) found that the numerical magnitude of numbers presented centrally before a simple stimulus detection task had a systematic impact on participants’ performance. Participants responded faster to left-sided targets than to right-sided targets when targets followed the presentation of small numbers. An equivalent advantage was observed for right-sided stimuli following large numbers. Similarly, Nicholls et al. (2008) demonstrated that participants were biased in their decision about which of two gray scales was darker by the numerical magnitude of superimposed digits. Left- and right-ward attentional biases were observed for low and high numbers, respectively. In the line-bisection effect, participants are relatively accurate at finding the midpoint of a line comprised of a series of “x”s, but deviate left- or right-ward when the line is comprised of a string of the word “two” or “nine,” respectively (Fischer, 2001; Calabria and Rossetti, 2005). Finally, damage to right parietal cortex elicits visuo-spatial hemineglect alongside representational neglect of portions of the MNL (Zorzi et al., 2002). Patients suffering from hemineglect not only misperceived visual information from the left hemifield, they also neglected numerical information from the left side of the MNL; when asked for the numerical middle between 1 and 9 the patients responded “6,” as if they did not consider the smaller numbers located on the left side of the mental number representation.

Together, the above findings strongly support the notion of a left-to-right oriented and logarithmically compressed MNL. This construct is central when considering the nature of the mechanisms put forth to explain the phenomenon of OM. Here we will detail three such proposed mechanisms, which are not necessarily exclusive of each other. The first mechanism was proposed by McCrink et al. (2007) in their original documentation of OM, and implemented in a computational model by Chen and Verguts (2012). It is based on two notions: first, it assumes a compressed mental magnitude representation. Second, it assumes that the cognitive system “undoes” the compression during mental calculation and operates on uncompressed magnitudes. This process of uncompression may be subject to a systematic bias which results in a slightly compressed magnitude code during calculation. This compressive bias may in turn cause the OM. A simple example illustrates this idea. Imagine a participant adds two numbers, e.g., 20 + 5. Internally, these are represented as log10(20) = 1.301 and log10(5) = 0.699. In the most extreme case, the uncompression process fails completely and participants will actually operate on the log-scaled values and add log10(20) = 1.301 and log10(5) = 0.699. Adding two logarithms corresponds to multiplying their linear-scaled values, i.e., log10(20 + 5) ~ 20 × 5 = 100 and in most cases this would result in values larger than the actual outcome. A similar argument holds for subtraction which would be replaced by division. Note that this example is used only to illustrate the basic idea of this account. The actually observed biases are much smaller than in this example. The main idea is that participants apply a linear transformation on a compressed scale which will lead to over- or under-estimating the outcome of a given problem. This hypothesis will be referred to as the “compression account.”

The second account appeals to attentional shifts along the MNL. According to this hypothesis, arithmetic operations are mediated by a dynamic interplay between cortical structures which process spatial information. In particular, it has been reasoned that a bilateral circuitry involving posterior superior parietal lobe (PSPL) and horizontal intraparietal sulcus (hIPS) that implements a form of vector addition over eye and retinal position information is co-opted by mental arithmetic. Indeed, exploiting the fact that saccades are accompanied by shifts of spatial attention in saccade direction, Knops et al. (2009a) used the brain activity elicited by left- and right-ward saccades to predict whether French-speaking participants were performing centrally presented addition or subtraction problems. The authors found that addition problems corresponded to the neural activity associated with right-ward saccades, presumably since participants shift attention toward larger numbers on the right side of the MNL. OM results from the momentum that drives participants too far along the MNL in the direction of the operation. This hypothesis will be referred to as the “attentional shifts account.”

Finally, a third hypothesis was proposed by McCrink and Wynn (2009) to explain the possible presence of OM in a population of 9-month-old infants. The authors presented visual sequences of addition and subtraction problems using non-symbolic numerosities (such as 6 + 4 = 5, 10, or 20), and provided the infants with three different types of outcomes to these problems: correct, too large, or too small. The infants looked reliably longer to the outcomes that violated the “momentum” of the particular problem. Infants who saw an addition scenario looked for a relatively long time at outcomes that were too small, but similarly to outcomes that were correct and too big; infants who saw a subtraction scenario looked longer at outcomes that were too large, and less to the correct and too-small outcomes. The authors put forward two suggestions to explain this pattern, the first was a computational account in which the infants are genuinely computing an acceptable outcome with some amount of error in magnitude representations that went in the “direction” of the operation. However, since a full-fledged, spatially oriented MNL is unlikely in this relatively unenculturated population, the authors hypothesized that they may instead be deploying general arithmetic principles. Specifically, “if adding, accept more” than the original operand, and “if subtracting, accept less (McCrink and Wynn, 2009, p. 407).” We will refer to this hypothesis as the “heuristics account.”

No study to date has attempted to disentangle these alternatives. In the following experiment we systematically examine potential determinants of OM, looking at a population that serves as a transition group between infants and adults-children in their first year of school. Six- and seven-year-old children were given a series of non-symbolic addition and subtraction problems, in which they had to choose the correct outcome from an array of several possible outcomes. The children were placed in either a reading condition or a cueing condition, in which we also separately tested their reading automaticity or attentional orienting capacity, respectively. (A group of adults was also tested on the non-symbolic addition and subtraction task, to ensure the efficacy of the paradigm in eliciting OM.) This design allows us to address two outstanding questions in the literature. First, do we see any evidence for a MNL at this age, as exhibited by the overall presence of a spatial–numerical interaction during arithmetic operations (OM)? Second, insofar as they exhibit OM, what are the determinants of the presence of this phenomenon?

Our predictions are as follows. First, if the MNL is instantiated via the highly automatic and culturally directed reading habits of the children, we will see a positive relationship between reading fluency and level of exhibited OM. Reading has been shown to modulate number-space associations while illiterate individuals did not exhibit consistent associations between numbers and space (Shaki et al., 2012). If there is not a spatially organized MNL at this age, regardless of reading ability, or if the MNL is present but not dictated by the child’s literacy, we will observe no relationship between these two constructs. Second, if OM is due to the flawed uncompression of mental numerosities during the course of mental arithmetic, we should observe a standard adult-like OM effect in 6- and 7-year olds, irrespective of their measures on reading or attention. Ample evidence from line-bisection tasks supports a logarithmically compressed magnitude representation early in formal schooling, with only a prolonged shift to linear representations culminating in sixth grade – and then only for scales that have become familiar (Siegler and Opfer, 2003; Opfer and Siegler, 2007; Barth and Paladino, 2011). The distribution of responses should resemble the pattern shown by adults and peak at or around the correct outcome with a higher degree of acceptance of over- or under-estimated outcomes (for addition and subtraction, respectively). There will be a fall-off as the incorrect outcomes become extremely discrepant from the correct outcomes. Third, if the children’s responses are driven by a “if adding, accept more, if subtracting, subtract less” heuristic account, we would expect to find an OM bias whose effect size would resemble the effect size found in adults. However, since children would not be engaging in an approximate calculation process, but rather show a general tendency to choose larger outcomes for addition and smaller outcomes for subtraction, the distribution of responses would differ from what is observed in adults. The distribution would not necessarily be centered on or around the correct response in a given set of presented response alternatives. Rather, we should see a trend to accept as correct any amount larger than the first operand (for addition) or smaller than the first operand (for subtraction). Again, we would expect no effect of reading ability or attentional indices if this pattern was observed. Finally, if OM is underlain by shifts of spatial attention along a MNL, we will see a relationship between attentional indices and the strength and/or presence of OM. Children who exhibit strong orienting responses in the presence of spatial cues may have more adult-like circuits for deploying attention, and this results in adult-like OM in which *these particular children* peak in response choice relatively close to the correct answer, with a margin of acceptance for somewhat larger outcomes during addition problems and somewhat smaller outcomes for subtraction problems. At least two attentional functions can be distinguished that are relevant in the present context. Attentional selection refers to the observed benefit in performance in response to stimuli appearing at attended locations. When a stimulus appears at unattended locations, however, attention has to be re-oriented toward the formerly unattended location. This reorientation is time-consuming and hence responses to stimuli at unattended locations are delayed. Neuroimaging studies point to distinct cortical circuits for selection and reorienting (Corbetta et al., 2008).

## Materials and Methods

### Participants

#### Children

The final sample consisted of 32 children (15 boys, 17 girls) between 6 (*n* = 15) and 7 (*n* = 17) years of age [mean (SD) age: 6.5 (0.51) years]. An additional four children were tested but excluded due to refusal to complete the study (3) or to homogenize the age range of the final sample (1). We also had an exclusion criterion for any children who were fluent in a right-to-left language (e.g., Hebrew); no one tested met this criteria. These children were recruited in the greater NYC area via word of mouth or through a local school. Informed written consent from the parents was obtained for all tested children. All children participated in a non-symbolic arithmetic task. Half of the sample (16) additionally participated in an attentional cueing paradigm [mean (SD) age: 6.38 (0.5) years], and the other half participated in a reading test [mean (SD) age: 6.69 (0.48) years]. In order to eliminate any interactions between the primary and secondary tasks, the order of the tasks was counterbalanced such that half the children received the primary adding/subtracting task first, and the other half received the secondary attention/reading task first.

#### Adult controls

A final sample of 32 college students (4 males, 28 females), recruited via the introductory psychology subject pool at a NYC university, were tested with the same non-symbolic calculation paradigm as the children. An additional eight students were excluded, seven for fluency in a right-to-left language (e.g., Hebrew), and one to keep sample size comparable between both groups. All students gave written informed consent and had normal or corrected-to-normal sight. Mean age was 18.88 years (SD = 2.06).

### Tasks

#### Arithmetic task

##### Stimuli

Four addition and four subtraction problems were created. Problems are presented in Table 1. To evaluate the differential effect of the arithmetic operation irrespective of numerical size of the outcome, we chose problems such that both arithmetic operations covered the same numerical range of final outcomes. Additionally, three memory trials were created with a second operand of zero to assess the overall capacity to retain the shown numerosities in mind.

**Table 1:** **Arithmetic problems presented in the non-symbolic calculation experiment and their correct and deviant results**.

Operands		Results and deviant response alternatives								
First	Second	1/2	1/1.7	1/1.4	1/1.2	1	1.2	1.4	1.7	2
**ADDITION**										
6	2	4	5	6	7	8	10	11	13	16
6	4	5	6	7	8	10	12	14	17	20
14	5	9	11	13	16	19	23	27	32	38
14	11	13	15	18	21	25	30	35	42	50
**SUBTRACTION**										
16	8	4	5	6	7	8	10	11	13	16
16	6	5	6	7	8	10	12	14	17	20
32	13	9	11	13	16	19	23	27	32	38
32	7	13	15	18	21	25	30	35	42	50
**MEMORIZATION**										
6		2	3	4	5	6	7	8	10	12
19		9	11	13	16	19	23	27	32	38
25		13	15	18	21	25	30	35	42	50

Apart from the correct result, eight deviant results were created for each arithmetic problem. These deviants were arranged as a geometric series (i.e., were linearly spaced on a logarithmic scale) and ranged from double the correct result to half of the correct result [technically, they were generated as round (*c* × 2*^*i*^/4*), where *c* is the correct result and *i* ranges from −4 to +4]. Since this procedure would lead to some identical response alternatives for memory trials with set size = 6 (rounded deviants: [3, 4, 4, 5, 7, 8, 10, 12]) due to rounding to integers we repeatedly subtracted one from the smallest duplicate deviant until no further deviants were present. This resulted in the following response alternatives: [2, 3, 4, 5, 7, 8, 10, 12]. To avoid a strategy of always selecting the response falling in the middle of the proposed range, only six out of those nine possible results were presented on screen. In 50% of the trials we presented the upper six (high range), and thus the correct result was the second smallest numerosity (although numerosities were randomly mixed). In the other 50% of the trials the lower six choices were shown (low range), and the correct result was therefore the fifth smallest numerosity. For example, for a problem such as 6 + 2 = 8 the low range would correspond to response alternatives 4, 5, 6, 7, 8, and 10. The high range would correspond to response alternatives 7, 8, 10, 11, 13, and 16.

To prevent the use of non-numerical cues, the sets of dots representing the non-symbolic numerosities were designed and generated using Matlab^®^ such that dot size changed, but total dot area in a given set was always fixed across stimuli for half of the trials. As a result of this manipulation, average item size covaried inversely with numerosity during the presentation of the operands (i.e., sets with smaller numerosities had larger dots). For the second half of the trials individual dot size was held constant and total dot area covaried with numerosity (i.e., sets with smaller numerosities had smaller total dot area). Thus, neither total occupied area nor individual dot size could serve as a cue for distinguishing between the different numerosities throughout the experiment. To avoid memorization effects due to repetition of a particular stimulus, on each trial stimulus images were randomly chosen from a set of 10 precomputed images with the given numerosity.

### Procedure

**Figure 1:**
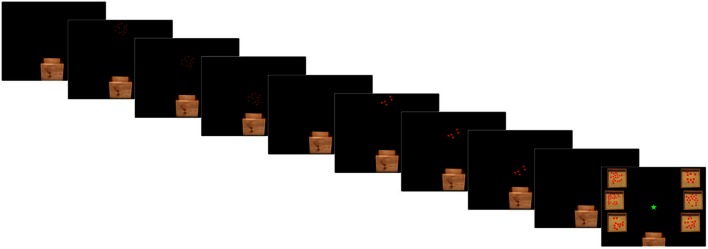
**Screenshots taken during an addition trial**. Two dot clouds sequentially move from the top of the screen into the box before the inside of the box is shown with six response alternatives. Children were asked to indicate the correct outcome by clicking on the respective box.

The non-symbolic calculation task contained addition, subtraction, and memory trials. Each trial started with the presentation of a picture of a monkey that disappeared after a mouse button was clicked. An empty wooden box appeared at the bottom of the screen and the first set of dots moved into the box. The dot set appeared at the top of the screen and moved toward the middle of the screen with decreasing speed such that it would briefly remain stationary at the screen center before speed increased again and the dot disappeared inside the box. For addition problems a second set of dots appeared on screen and disappeared inside the box in the described manner. For subtraction problems a set of dots moved out of the box and disappeared at the top of screen. For memory trials only one set of dots disappeared in the box. After all sets of dots disappeared, three response alternatives appeared on the left and right side of the screen in an elliptic fashion, randomized for spatial location (i.e., the quantities are randomly assigned to the six positions). Each response alternative was presented as the top view of the box which contained different numbers of dots (see above). Children were asked to choose the correct outcome by clicking on the respective box. The beginning of the response active period was indicated by the appearance of the mouse pointer on top of a green star in the center of the screen. See Figure 1 for a depiction of an exemplar addition trial. A total of 44 trials were presented, 12 memory trials, 16 addition, and 16 subtraction trials. A training period consisting of eight trials preceded the actual paradigm. In the training period, responses were not time-limited and feedback was provided. A correct choice was indicated by a green frame around the chosen box. The appearance of a red frame around the chosen box indicated that the choice was incorrect. During training response alternatives remained on screen until the correct response alternative was chosen. For the test period we limited response time to a maximum of 20 s. In the testing period, no feedback about the correctness of the choice was provided. The chosen alternative was highlighted by a surrounding blue frame, irrespective of correctness. On average children completed all calculation trials in about 20 min.

#### Attention task

##### Stimuli and procedure

An adapted Posner paradigm was administered to half of the child sample. A capital letter X in the center of the screen served as fixation point. On each trial the fixation was replaced by a yellow smiling face after a random interval between 1200 and 2400 ms. The smiling face indicated a forthcoming trial and remained visible for 750 ms. It was accompanied by an acoustic signal that served to attract children’s attention. After a 250-ms interval with no stimuli on screen a blue arrow appeared in the center of the screen that pointed either to the left, to the right, or in both directions. The bidirectional arrow served as neutral condition. After a variable delay (200 or 800 ms) a blue star (introduced as “bug” to the children) appeared lateral to the arrow. The children were instructed to “zap the bug” by pushing the response button on the side of the target stimulus as fast as possible. The single headed arrows provided valid information about the side of the upcoming target in 67% (*n* = 40) of the trials (valid trials). In invalid trials (*n* = 10; 17%) the target stimulus appeared opposite the pointing direction of the arrow. The neutral condition was presented in 17% of the trials (*n* = 10). Target side (left, right) was balanced (50% each) in valid, invalid, and neutral trials. When the children hit the response button the target disappeared and an acoustic sound indicated whether the response was correct (sound 1) or not (sound 2). Eight training trials preceded the test and served to illustrate the task. The task lasted about 5–8 min.

This design allowed us to evaluate two central parameters of the attentional system: orienting (selection) and reorienting. Adopting common procedure from the attention literature the orienting effect was computed as the difference in reaction times between neutral and valid trials, the reorienting effect was computed as the difference in reaction times between neutral and invalid trials. All reaction time analyses were based on correct responses only.

#### Reading task

##### Stimuli and procedure

In order to assess reading ability and fluency, each child was provided with a developmentally appropriate vignette to read aloud while the experimenter tape-recorded him/her on the computer. The children were instructed to “Please read as much of this as you can, trying not to make any mistakes.” The vignette was as follows:

Mother and Father frog were going to a party. Mrs. Turtle came to babysit.

“Hello, little frogs,” said Mrs. Turtle, “What are we going to do tonight? Would you like me to read you a story?”

“Yes! Yes!” said the little frogs, “we would like that very much.”

Mrs. Turtle finished reading. The little frogs cried, “Would you like to jump with us?”

“Not now,” said Mrs. Turtle, “It’s suppertime. I will make you a nice supper.”

“Ok!” said the little frogs, “we are very hungry.”

A coder blind to the hypotheses of the experiment reviewed the tapes of the children and assessed the subjective relative level of fluency for each child (from 1 to 10), and also coded how long each child took to read the vignette. Because we found that the time-to-read measure captured fluency in a more objective way than the coder rating, we used those data as each child’s fluency score in our analyses.

### Data analysis

Data were analyzed using SPSS^®^ under a classical statistical null hypothesis significance testing (NHST) approach. Bayesian analyses were conducted using R (R Development Core Team, 2010).

## Results

### Children

#### Arithmetic task

##### Can children memorize the operands?

**Figure 2:**
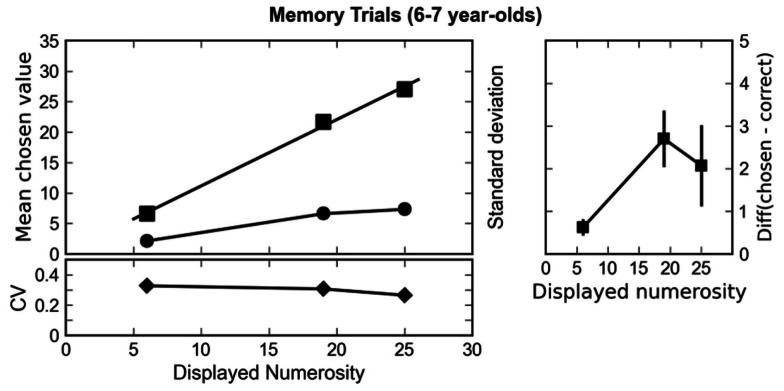
**Left column: mean responses (chosen values, squares) of the subjects and standard deviations (circles) plotted against the correct outcome for memory trials**. The lower part depicts the coefficient of variation (CV, diamonds) – that is, the ratio of standard deviation and mean chosen value, plotted against the displayed numerosity. A constant coefficient of variation indicates that variability of chosen values increased proportionally with mean chosen value. In turn, this can be understood as an instantiation of Weber’s law. Right column: the difference between the displayed numerosity and the chosen value plotted against the displayed numerosity. Positive values indicate that children tended to overestimate the remembered numerosities. Error bars indicate the standard error of the mean.

First, we demonstrate that children were indeed capable to process and memorize the presented numerosities, albeit in an approximate fashion. If so, the mean chosen value on memory trials should closely follow the presented numerosities. Figure 2 shows that this is actually the case. Mean chosen numerosity (squares) increased significantly with presented numerosity [*F*(2, 60) = 279.65, *p* < 0.001, epsilon = 0.88 (Huynh and Feldt, 1970)]. This main effect did not interact with age [*F*(2, 60) = 1.17, *p* = 0.32], indicating that both age groups were capable of remembering the presented numerosities. In line with the assumption of the mental magnitude representation following Weber’s law we observed a constant coefficient of variation that did not significantly covary with numerosity [*F*(2, 60) = 2.20, *p* = 0.12; lower part of Figure 2]. No significant interaction with age was observed [*F*(2, 60) < 1]. A slight tendency to overestimate the remembered numerosities was present in the data. Figure 2 (right) depicts the difference between mean chosen numerosity and the memorized numerosity. To test whether this difference was statistically different from zero we computed a linear regression (*y* = *a* + *bx*) to predict memorized numerosity (*y*) based upon shown numerosity (*x*). If children were systematically overestimating the numerosities the intercept (*a*) of this regression equation would be significantly larger than zero. Mean intercept (*a* = 0.63) did not differ significantly from zero [*t*(31) = 1.72, *p* = 0.095]. However, mean slope (*b* = 0.28) was significantly larger than zero [*t*(31) = 8.65, *p* < 0.001], indicating that estimates increased with shown numerosity.

##### Did children engage in approximate calculation or respond at random?

**Figure 3:**
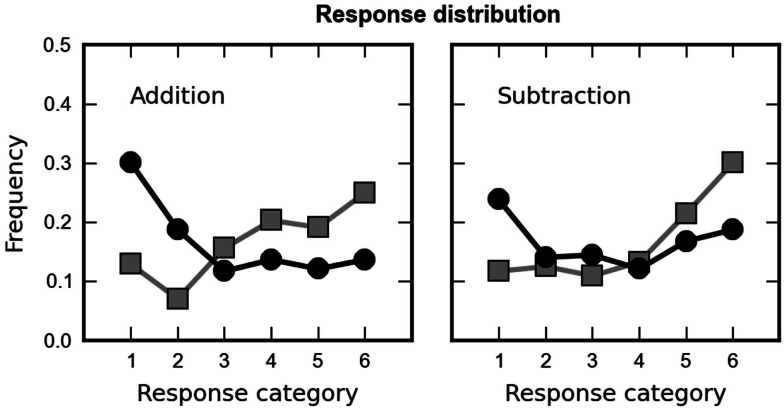
**Distribution of the children’s choices across the six proposed results, averaged over all arithmetic problems, separately for addition (left column) and subtraction (right column)**. The children’s responses were not distributed randomly but depended on the range of response alternatives presented (high or low range shown as black squares and gray squares, respectively). Responses were centered around the values that were closest to the correct outcome (fifth for low range and second for high range).

Next, we analyzed whether the subjects chose among the proposed choices at random. On each trial, six response alternatives were presented. They were either sampled from the lower range of response alternatives (alternatives one through six in Table 1) or the upper range of response alternatives (alternatives 3 through 9 in Table 1). As a consequence the correct outcome was either the second (high range) or the fifth smallest response alternative (low range) on screen. If the subjects were able to solve the arithmetic problems, their response choices should show a non-flat distribution, presumably centered close to the correct value. In contrast, if they responded randomly, we would not expect any differences in the frequency of choosing a particular response alternative. In Figure 3, we plot response frequency for each operation, separately for trials in which the correct answer was second (black) or fifth (gray). Responses were clearly distributed non-randomly. The peak of the distribution was always centered on response alternatives close to the correct outcome.

These conclusions were supported by an analysis of variance (ANOVA) over the different response categories, with (arcsine-transformed) percentage of choice as the dependent variable and rank of the subject’s choice (one to six), range (second or fifth value correct), and operation (addition, subtraction) as factors. A main effect of rank [*F*(5, 155) = 3.80, *p* = 0.012, epsilon = 0.62] was observed, indicating an unequal distribution of response frequencies and therefore speaking against a random choice pattern. Most importantly, a significant interaction between rank and range [*F*(5, 155) = 10.36, *p* < 0.001] was observed, indicating that children indeed chose values close to the correct outcome. No other main effects or interaction were significant. The absence of significant interactions between rank and operation or between all three factors indicates that this response pattern was comparable for both arithmetic operations.

##### Did children’s performance in the non-symbolic calculation task conform to Weber’s law?

**Figure 4:**
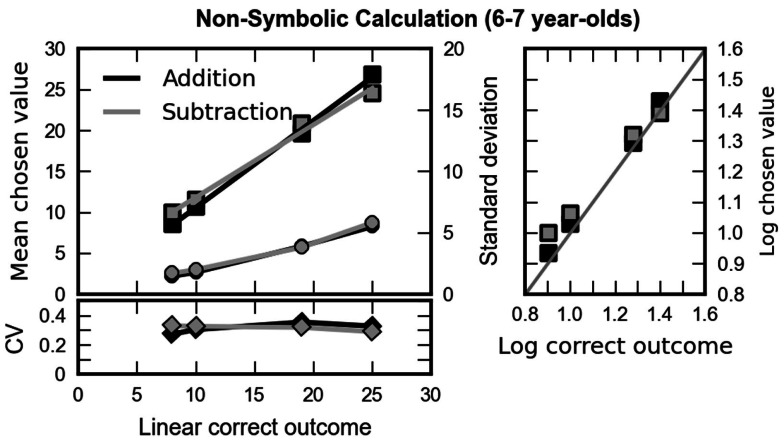
**Left column: mean responses (chosen values, squares) of the children and standard deviations (circles) plotted against the correct outcome for addition (black) and subtraction problems (gray)**. The lower part depicts the coefficient of variation (CV, diamonds) – that is, the ratio of standard deviation and mean chosen value, plotted against the correct outcome. A constant coefficient of variation indicates that variability of chosen values increased proportionally with mean chosen value. In turn, this can be understood as an instantiation of Weber’s law. Right column: the logarithm of the correct outcome plotted against the logarithm of the mean value chosen by the children for addition (black) and subtraction (gray). The gray line indicates a ratio of 1 – that is, perfect performance.

We next examined how children responded to our different arithmetic problems. The left column of Figure 4 shows the children’s mean responses (chosen values) as a function of the size of the correct result, separately for the two operations. If the children were able to solve the arithmetic problems, the chosen value should increase as a function of the correct outcome. With increasing numerical magnitude, theory predicts an increasing variability of the chosen values (see the appendix in Barth et al., 2006). Finally, according to Weber’s law, the increase in the chosen values should be paralleled by a proportional increase in response variability, as expressed in terms of their respective standard deviation, resulting in a constant coefficient of variation (CV, the ratio of the standard deviation, and mean of the subjects’ responses) across arithmetic problems of different numerical magnitude. As can be seen in Figure 4, both children’s mean responses (depicted as squares) and their standard deviation (depicted as circles) increased as a function of the correct outcome for both addition (black) and subtraction (gray). This impression was confirmed by repeated measures ANOVAs of mean and standard deviation with operation (addition, subtraction) and numerosity (8, 10, 19, 25) as within-group factors and age as between group factor. Mean responses [*F*(3, 90) = 313.04, *p* < 0.001, epsilon = 0.60] and variability [*F*(3, 90) = 53.70, *p* < 0.001, epsilon = 0.68] of responses increased significantly with increasing correct outcome. Interestingly, the increase of mean chosen value and its variation were stronger for addition than for subtraction as indicated by the significant interactions [mean: *F*(3, 90) = 6.42, *p* = 0.001, epsilon = 0.82; SD: *F*(3, 90) = 3.12, *p* = 0.035, epsilon = 0.91]. No other main effect or interaction was significant.

As can be seen in the lower left part of Figure 4, the CV was constant across the whole range of outcomes for addition and subtraction. This was tested statistically with a repeated measures ANOVA with operation (addition, subtraction) and numerosity (8, 10, 19, 25) as within-group factors and age as between group factor. No main effect or interaction reached statistical significance (minimum *p* = 0.133). To further corroborate this finding we calculated the difference between the correct outcome and the mean chosen value, once both of them had been transformed to a logarithmic scale, and calculated a repeated measures ANOVA on the standard deviations of these differences, with size of the correct result as the only factors, separately for both operations (addition and subtraction). Neither for addition [*F*(3, 93) = 2.65, *p* = 0.053] nor for subtraction [*F*(3, 93) = 0.977, *p* = 0.407] we observed a significant impact of problem size on the standard deviation of this index.

Taken together, these results suggest that data are well described by Weber’s law which is in line with the assumption that the underlying mental magnitude representation is logarithmically compressed. Therefore, all following analyses concerning the OM effect were carried out in a logarithmic scale, using as input the difference between the logarithm of the correct outcome and the logarithm of the chosen value. Such analyses also have the advantage of more likely meeting the prerequisites of the ANOVA, which stipulates that all data have a fixed variability.

##### Did children show operational momentum in non-symbolic calculation?

**Figure 5:**
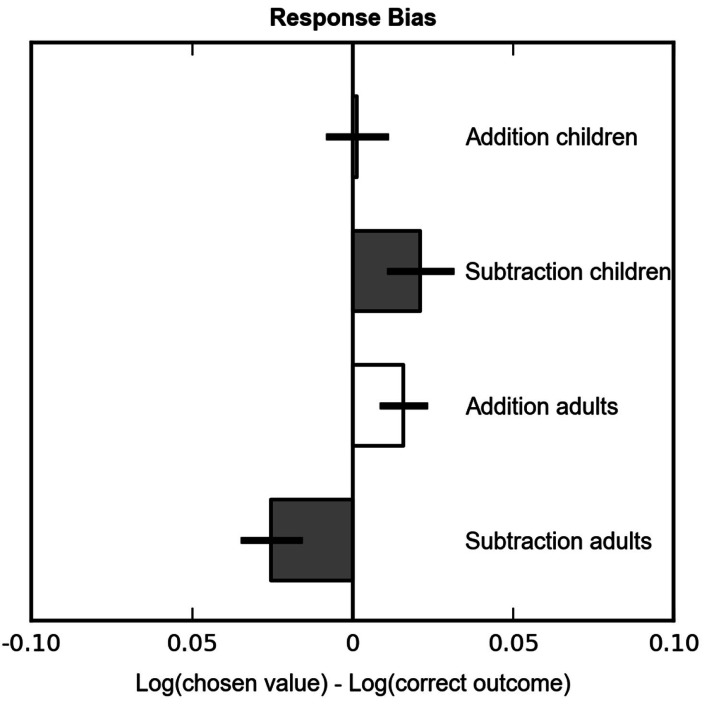
**Mean response bias for children and adults, defined as the difference between the chosen value and the correct outcome, both expressed on a log scale**. A negative bias indicates underestimation, and a positive bias indicates overestimation.

To quantify this OM effect, we computed a simple estimate of response bias: the mean difference between the log of the subject’s responses and the log correct result. This value was submitted to an ANOVA with operation as within-group factor and age as between group factor. Most importantly, no main effect of operation was observed [*F*(1, 30) = 3.02, *p* = 0.092], that is, no significant bias toward smaller responses for subtraction than for addition was observed for children. No significant interaction with age was observed [*F*(1, 30) = 0.76, *p* = 0.39]. Results are shown in Figure 5.

#### Cueing task

Overall performance in the cueing task was very good. Children committed only a total of 20 errors corresponding to 2% that were excluded from all subsequent analyses.

Mean reaction times from valid, invalid, and neutral conditions were computed per child and *z*-standardized per child (mean = 0, SD = 1) to account for high between-subject variability in reaction times from children.

##### Benefit and cost in the cueing task

**Figure 6:**
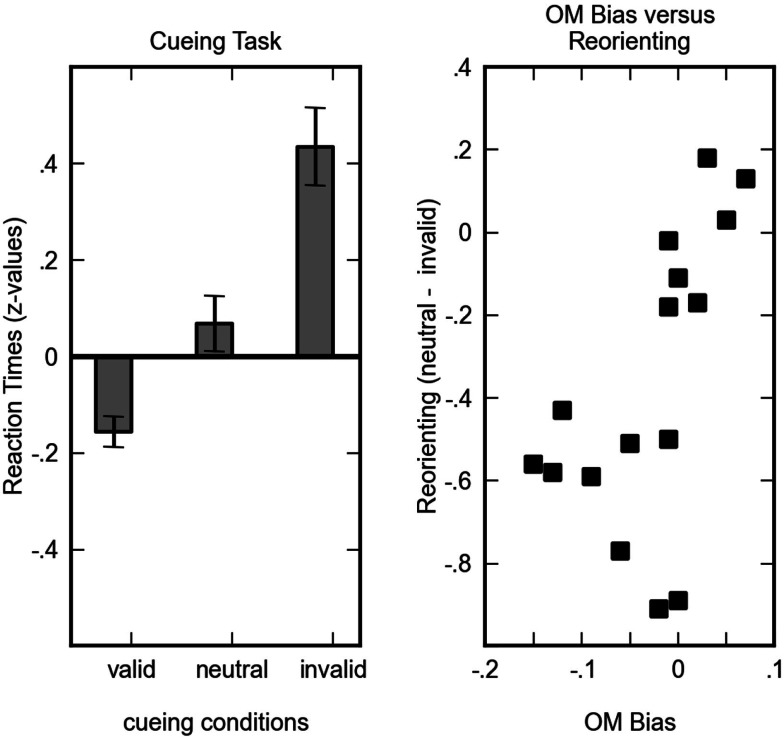
**Left column: *z*-standardized mean reaction times for valid, neutral and invalid conditions of the attention paradigm**. Error bars indicate standard error of the mean. Right column: the reorienting effect (difference between neutral and invalid trials) plotted against the operational momentum bias. For reorienting better performance is indicated by numerically larger (i.e., less negative) values. A regular operational momentum effect corresponds to positive values, an inverse operational momentum effect corresponds to negative values. The correlation between reorienting and operational momentum signifies that the less children suffer from invalid cueing the more they are prone to exhibit a regular operational momentum effect.

First, we analyzed the effects of cueing on reaction times by computing the benefits and the costs of valid and invalid trials with respect the neutral condition, respectively. Adopting standard nomenclature from attention domain the benefit (neutral – valid) will be referred to as orienting effect and cost (neutral – invalid) will be referred to as reorienting effect. Figure 6 (left) depicts the cueing effects and implies that the type of cue (valid, invalid, or neutral) had a measurable impact on children’s performance. This impression was supported by a significant main effect of cue type in a repeated measures ANOVA with cue type (valid, invalid, neutral) and SOA (200 ms, 800 ms) as factors [*F*(2, 30) = 21.13, *p* < 0.001]. No other main effect or interaction was significant, implying that the observed cueing effects were not statistically modulated by SOA. Paired *t*-tests revealed a significant orienting effect {valid trials were responded to faster than neutral trials [*t*(15) = −2.72, *p* = 0.016]} and a significant reorienting effect {neutral trials were responded to faster than invalid trials [*t*(15) = −4.16, *p* = 0.001]}.

#### Correlating cueing effects with calculation data

It has been argued that the OM effect is at least partially due to attentional shifts induced by the arithmetic operation that is operating on a spatially oriented mental magnitude representation. According to this account additions are associated with attentional shifts to the right and subtractions are associated with attentional shifts to the left. To test this account we assigned half of the children in the current study to a cueing paradigm. If the OM effect is a consequence of the interaction between the attentional systems and the mental magnitude system we should observe a correlation between both measures over children. That is, children with a large OM effect should also exhibit larger attentional cueing effects. To test this account we computed Pearson correlation coefficients between the OM bias (that is [log(chosen_Addition_) − log(correct_Addition_)] − [log(chosen_Subtraction_) − log(correct_Subtraction_)] and the orienting and reorienting effects observed in the cueing paradigm. Note that this analysis, too, is based upon correct trials in the cueing paradigm only. While orienting did not correlate significantly with OM bias (*r* = 0.14, *p* = 0.616), a significant correlation between OM bias and reorienting was observed (*r* = 0.59, *p* = 0.017). The difference between invalid and valid trials did not significantly correlate with OM bias (*r* = −0.37, *p* = 0.158). In Figure 6 (right) we plot the individual OM biases against the reorienting effect. It becomes evident that the relatively high correlation was not driven by few outliers but that over the entire range of reorienting and OM bias a higher OM bias was associated with smaller reorienting effects, that is with lower costs due to invalid cueing.

#### Correlating reading fluency with OM

We hypothesized that reading may corroborate existing spatial–numerical links and may thus be linked with OM. To test this idea we assessed individual reading capacities by measuring reading durations for a short text. Although reading durations showed substantial variability (mean = 114 s; SD = 94 s) no correlation was observed with OM [*r*(reading, OM bias) = −0.18, *p* = 0.513].

### Adults

To test whether the current version of the paradigm is suited to reveal OM effects we administered the same paradigm to a group of 32 students from Barnard College. The same analysis steps as for children were performed for adults and described briefly below.

#### Can participants memorize the operands?

Again we start by testing whether participants were able to correctly memorize the shown values. Both mean chosen values (6.7, 20.8, and 25.4 for displayed numerosities 6, 19, and 25, respectively) and the standard deviation of chosen memorized value increased significantly with presented numerosity [mean: *F*(2, 62) = 473.14, *p* < 0.001, epsilon = 0.83; mean: *F*(2, 62) = 38.28, *p* < 0.001] indicating that participants were capable of remembering the presented numerosities. In line with the assumption of the mental magnitude representation following Weber’s law we observed a constant coefficient of variation that did not significantly covary with numerosity [*F*(2, 60) = 1.33, *p* = 0.273, epsilon = 0.88].

#### Did participants engage in approximate calculation or respond at random?

**Figure 7:**
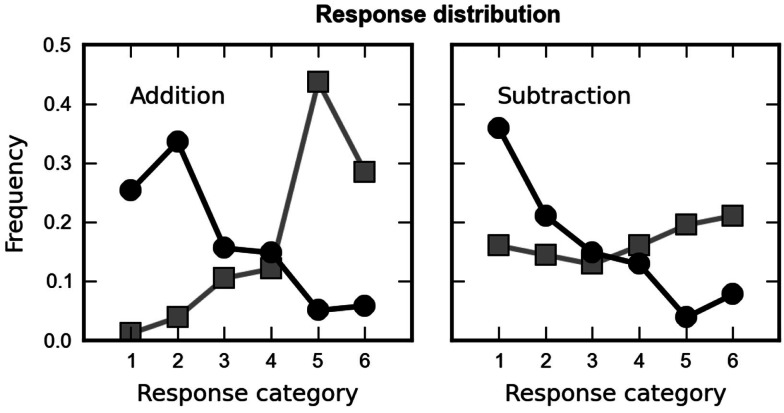
**Distribution of the participants’ choices across the six proposed results, averaged over all arithmetic problems, separately for addition (left column) and subtraction (right column)**. Responses were not distributed randomly but depended on the range of response alternatives presented (high or low range shown as black squares and gray squares, respectively). Responses were centered around the values that were closest to the correct outcome (fifth for low range and second for high range).

Next, we analyzed whether the subjects chose among the proposed choices at random. To this end we analyzed (arcsine-transformed) percentage of choice as the dependent variable in an ANOVA and rank of the subject’s choice (one to six), range (second or fifth value correct), and operation (addition, subtraction) as factors. A main effect of rank [*F*(5, 155) = 3.57, *p* = 0.006, epsilon = 0.89] was observed, indicating an unequal distribution of response frequencies and therefore speaking against a random choice pattern. Most importantly, a significant interaction between rank and range [*F*(5, 155) = 54.14, *p* < 0.001] was observed, indicating that participants did not engage in a random choice pattern. Operation interacted significantly with range [*F*(5, 155) = 4.22, *p* = 0.049], rank [*F*(5, 155) = 6.84, *p* < 0.001, epsilon = 78], and with range and rank [*F*(5, 155) = 9.88, *p* < 0.001). Results are shown in Figure 7.

#### Did participant’s performance in the non-symbolic calculation task conform to Weber’s law?

**Figure 8:**
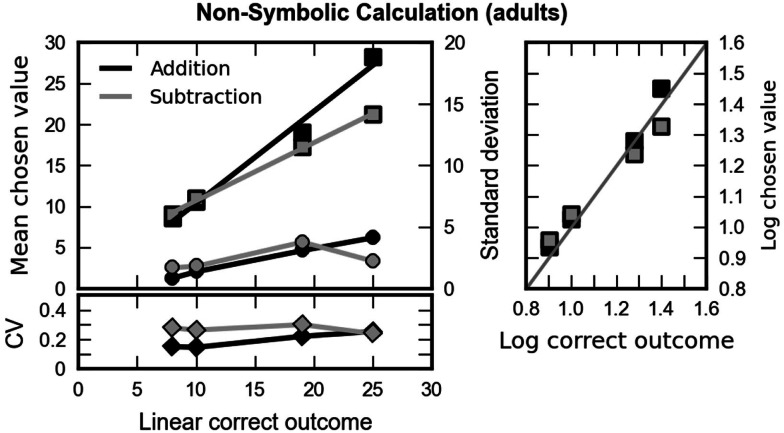
**Left column: mean responses (chosen values, squares) of the subjects and standard deviations (circles) plotted against the correct outcome for addition (black) and subtraction problems (gray)**. The lower part depicts the coefficient of variation (CV, diamonds) – that is, the ratio of standard deviation and mean chosen value, plotted against the correct outcome. A constant coefficient of variation indicates that variability of chosen values increased proportionally with mean chosen value. In turn, this can be understood as an instantiation of Weber’s law. Right column: the logarithm of the correct outcome plotted against the logarithm of the mean value chosen by the participants for addition (black) and subtraction (gray). The gray line indicates a ratio of 1 corresponding to perfect performance.

We next examined how the subjects responded to the different arithmetic problems. Specifically, repeated measures ANOVAs with numerosity and arithmetic operation revealed that: (a) chosen values increased as a function of correct outcome [*F*(3, 93) = 764.36, *p* < 0.001, epsilon = 0.85], (b) the variability of the choices increased with increasing correct outcome [*F*(3, 93) = 73.19, *p* < 0.001, epsilon = 0.86]. We found that the coefficient of variation increased with increasing correct outcome [*F*(3, 93) = 3.82, *p* = 0.017, epsilon = 0.87]. A significant interaction between operation and numerosity [*F*(3, 93) = 6.15, *p* = 0.001] was due to the fact that the increase was present only for addition [*F*(3, 93) = 9.44, *p* < 0.001] but not for subtraction [*F*(3, 93) = 1.63, *p* = 0.188]. This pattern of results was corroborated by the results of two repeated measures ANOVAs on the standard deviation of the difference between the chosen values and the correct outcomes after they had been transformed to log scale with size of the correct outcome as the only factor. We observed a significant main effect of size for addition problems [*F*(3, 93) = 10.56, *p* < 0.001] but the size of the outcome did not systematically influence response variability for subtraction [*F*(3, 93) = 1.74, *p* = 0.164]. Results are shown in Figure 8.

#### Did participants show operational momentum in non-symbolic calculation?

Finally, we analyzed the OM effect by computing the same bias as for children {[log(chosen value) − log(correct outcome)]}for each problem and averaged over addition and subtraction problems, separately. A paired sample *t*-test revealed a significant OM effect [*t*(31) = 2.91, *p* = 0.007] that took the form of a full cross-over effect. That is, participants significantly overestimated results for addition problems [*t*(31) = 2.12, *p* = 0.042] and under-estimated results for subtraction problems [*t*(31) = −2.61, *p* = 0.014]. Results are depicted in Figure 5.

### Joint analysis of OM in adults and children

To statistically test the observed discrepancy of regular OM in adults and the absence of a statistically significant OM in children we submitted the log-scaled bias [log10(chosen value) − log10(correct outcome)] from both groups to a common ANOVA with type of arithmetic operation (addition vs. subtraction) as within-subjects factor and group (adults vs. children) as between-subjects factor. Type of arithmetic operation did not have a significant impact on the observed calculation bias [*F*(1, 62) = 1.44, *p* = 0.234]. No main effect of group was observed [*F*(1, 62) = 2.601, *p* = 0.112]. Most importantly, in line with the observed discrepancy type of arithmetic operation significantly interacted with group [*F*(1, 62) = 11.62, *p* = 0.001], statistically corroborating the observation that the differential impact of the arithmetic operation on the chosen values depends on the group. Adults show a differential impact of arithmetic operation while children tend not to. This is in line with the observation that the OM bias for addition and subtraction is negatively correlated over adults (*r* = −0.343, *p* = 0.054) but positively correlated over children (*r* = 0.42, *p* = 0.017). Put differently, those adults who tend to larger overestimation in addition also tend to larger underestimation in subtraction. In children this pattern is reversed. Children who tend to larger overestimation in addition also show larger overestimation in subtraction.

### Joint analysis of OM in adults and children using a Bayesian approach

The repeated measures ANOVA model as implemented in SPSS assumes homoscedasticity, meaning that the variation of the dependent variable is the same for each experimental group and repeated measurement. In developmental research, this might however not be justified. If the comparison involves, for example, children, heteroscedasticity may be observed due to increased variation in functioning, compliance with the task, or both. Indeed, analyzing the standard deviation of the chosen values in a 2 (operation) × 4 (outcome) repeated measures ANOVA with age group (adults vs. children) as between group factor revealed a significant main effect of group [*F*(1, 62) = 23.15, *p* < 0.001], indicating that adults responses were less variable than children’s responses. Thus, the stipulated homoscedasticity cannot be assumed. As known from the statistical literature, heteroscedasticity can substantially decrease power (Wilcox et al., 1986; Wilcox, 1987), the probability of identifying an existing effect.

A straightforward way to overcome the implausible restriction of homoscedasticity is by using a Bayesian model with all (co)variances considered as unknown parameters. In the Bayesian approach, estimation is informed not only by information from the data (likelihood function), but also by *a priori* information (prior density function). Their product is proportional to the target of Bayesian estimation (posterior density). The posterior density is often computed with the help of Monte Carlo methods. For an introduction to the Bayesian approach for experimental researchers, see Kruschke and Safari Tech Books Online (2011).

Let *i* = 1, 2, …, 32, *j* = 1, 2, *k* = 1, 2 index subjects (1 through 32), measurements (addition vs. subtraction), and groups (adults vs. children), respectively. Then the data form an *i* × *j* × *k* array. We consider a multivariate normal likelihood. In probability notation, this can be put as:

(1) $$y_{\mathrm{ik}}\sim N\left(\mu_{k},\;\Sigma_{k}\right),$$

with the vector $y_{\mathrm{ik}}=\left(\begin{array}{c} y_{i1k}\\ y_{i2k} \end{array}\right)$ of the addition and subtraction scores of the *i*th subject from the *k*th group, $\mu_{k}=\left(\begin{array}{c} \mu_{1k}\\ \mu_{2k} \end{array}\right)$ the *k*th group’s means in addition and substraction, and $\Sigma_{k}=\left(\begin{array}{cc} \sigma_{11k}^{2} & \sigma_{12k}^{2}\\ \sigma_{21k}^{2} & \sigma_{22k}^{2} \end{array}\right)$ the group-specific covariance matrix. Notice that: (a) the model assumes correlated measurements as the secondary diagonal of **Σ*_k_*** is not restricted to zero and (b) it assumes heteroscedasticity as the variances are allowed to be unequal across groups and measurements, why we may consider it as a heteroscedastic repeated measures ANOVA model. Prior densities must be specified to complete the model. We choose a flat normal density for the measurement means of each group because we wish to let the data dominate the analysis:

(2) $$\mu_{\mathrm{jk}}\sim N\left(0,100000\right).$$

For the same reason, we assign the following inverse Wishart density to the covariance matrices with identity matrix $I=\left(\begin{array}{cc} 1 & 0\\ 0 & 1 \end{array}\right)$ and 2 degrees of freedom:

(3) $$\Sigma_{k}\sim \mathrm{inverse}-\mathrm{Wishart}\left(I,\;2\right).$$

Before running the model, data were standardized. We sampled μ_1*k*_, μ_2*k*_, $\sigma_{11k}^{2}$, $\sigma_{12k}^{2}$, and $\sigma_{22k}^{2}$ iteratively using JAGS 3.3.0. Convergence was checked by visual inspection. We discarded the first 100,000 out of 600,000 iterations as burn-in. Inference was based on the remaining. Marginal posterior densities including those of the effects of operation in adults and children, μ_11_ − μ_21_ and μ_12_ − μ_22_ respectively, and that of the interaction (μ_11_ − μ_21_) − (μ_12_ − μ_22_), are summarized in Table 2.

**Table 2:** **Marginal posterior densities from the Bayesian analysis**.

	Mean	HDI^a^
**MODEL PARAMETERS**		
Means
μ_11_	0.226	[−0.054, 0.502]
μ_21_	−0.513	[−0.863, −0.156]
μ_12_	−0.034	[−0.391, 0.324]
μ_22_	0.321	[−0.062, 0.702]
(Co)variances
$\sigma_{111}^{2}$	0.635	[0.353, 0.974]
$\sigma_{121}^{2}$	−0.265	[−0.589, 0.031]
$\sigma_{221}^{2}$	1.026	[0.571, 1.576]
$\sigma_{112}^{2}$	1.056	[0.582, 1.617]
$\sigma_{122}^{2}$	0.460	[0.054, 0.930]
$\sigma_{222}^{2}$	1.204	[0.666, 1.846]
**DERIVED QUANTITIES**		
Effects
μ_11_ − μ_21_ (addition vs. subtraction adults)	0.739	[0.221, 1.254]
μ_12_ − μ_22_ (addition vs. subtraction children)	−0.354	[−0.758, 0.048]
(μ_11_ − μ_21_) − (μ_12_ − μ_22_)	1.093	[0.450, 1.756]

^a^The highest density interval is a credible region, including the most likely values.

The probability that the effect is positive/negative in the light of the data is given by the appropriate integral of the marginal posterior density (Jackman, 2009). For μ_11_ − μ_21_, (μ_11_ − μ_21_) − (μ_12_ − μ_22_), and μ_12_ − μ_22_ this probability is 0.997, 0.999, and 0.958, respectively, 0.003, 0.001, and 0.042 are the probabilities of the contrary, namely that the effect is zero or less/more than zero. Considering their ratios, we conclude that beside decisive support for OM in adults and a difference in OM between adults and children, our data strongly supports the idea of an inverse OM in children.

## Discussion

In the current study we administered a non-symbolic calculation paradigm – along with a reading and an attentional cueing task – to 6- and 7-year olds to examine the presence and determinants of OM. Four main findings are of note. First, we replicate the presence of proficient non-symbolic addition and subtraction in an adult population, along with a systematic overestimation of addition outcomes, and underestimation of subtraction outcomes (McCrink et al., 2007; Knops et al., 2009b). Second, the children tested here were capable of non-symbolic addition and subtraction, using only their “number sense”; they reliably altered their responses to the offered outcomes to correspond with a mental calculation of the estimated correct outcomes, and did so without the aid of confounding perceptual cues. Third, while a group of adult controls showed a regular OM effect, children did not show a significant OM effect using classical statistical NHST. However, using Bayesian inference we observed a significant inverse OM effect. That is, subtraction problems lead to significantly larger overestimations than addition problems. Finally, there was a relationship between a child’s level of attentional reorienting and their propensity to exhibit regular OM; the lower the cost of the invalid cue the more regular OM bias exhibited by that child. Counter to hypotheses that offer self-directed automaticity of reading as a driver of spatial–numerical links, we found no relationship between reading fluency and OM.

What do the current findings implicate for the different hypotheses concerning the basis of OM and the developmental trajectory of OM?

### The compression hypothesis

The compression hypothesis assumes that the OM results from a flawed uncompression operation during the course of manipulating mental magnitudes (McCrink et al., 2007; Chen and Verguts, 2012). According to this hypothesis a regular OM effect was expected in the tested age range. The observed absence of an overall OM bias under classical statistical NHST in combination with a significantly reversed OM under Bayesian approach is hard to reconcile with this notion. One might argue that the compression of the MNL is not identical for adults and children at the age of 6–7 years, and therefore the OM should differ between adults and children. However, if anything compression of the mental magnitude representation is *more* pronounced for children in the tested age range, implying a regular OM bias that is even more pronounced than in adulthood (Siegler and Opfer, 2003; Opfer and Siegler, 2007). For example, Berteletti et al. (2012) found no significant differences between linear and logarithmic model for number to position task in first (mean age: 6;11) and second grade (mean age: 7;11). Preschoolers (mean age: 5;8) were best fit by logarithmic and as of third grade (mean age: 8;9) linear models provided best fit. Hence, the shift from logarithmic to linear mapping of numbers to positions occurs only in second or third grade, when children are older than the sample tested here. Therefore, the present results speak against a flawed compression-uncompression mechanism as the driving factor of the OM bias.

### The heuristics approach

The heuristics approach (McCrink and Wynn, 2009, p. 407) suggests that children deploy a general arithmetic principle of “if adding, accept more” than the original operand and “if subtracting, accept less.” According to this approach, too, an OM bias was expected for the tested age range (that is, given an addition and subtraction problem that yield the same objective answer, the average subjective outcome chosen as correct for addition will be higher than that chosen for subtraction). Moreover, the response distribution was expected to differ significantly from the distribution observed for adults. Under the strictest interpretation of this theory, if children had adopted such a heuristic rather than engaging in an approximate calculation they would have frequently chosen results that are larger than the first operand in addition and smaller than the first operand in subtraction, with no differentiation between somewhat vs. extremely larger/smaller. This would result in a response distribution that plateaus at results discriminably larger than the initial outcome and ranging up (addition) or those that start at any outcome discriminably smaller than the initial operand and go down (subtraction). The present study yielded two findings that speak against this heuristic approach. First, we did not observe an overall OM bias in 6- and 7-year-old children using NHST in combination with a significant inverse OM under Bayesian approach. Second, the response distributions did not follow the expected pattern under the assumption of a pure heuristic. Rather, the distributions largely resembled those observed for adult participants, with distinct response peaks (albeit less pronounced for subtraction problems). For example, for the high response range in addition problems the observed modal value was actually numerically smaller than the actual outcome. Together, these results imply that children did indeed engage in approximate calculation and speaks against the assumed heuristic of accepting generically “more” with addition or “less” with subtraction.

### Reading fluency account

The hypothesis that reading fluency underlies the formation of a spatial–numerical link, and its resultant OM bias, was also unsupported. Although there was a wide range of reading ability (ranging between 36 and 320 s to read through a short vignette) this factor did not correlate with a propensity to show regular OM. While numerous studies have found that the reading directionality of adults clearly modulates the traditional SNARC effect, it is likely not responsible for *instantiating* it (Shaki et al., 2009, 2012). In addition to the current findings, there are several studies which show the presence of other spatial–numerical relationships before the onset of reading (Opfer et al., 2010; Berteletti et al., 2012; Shaki et al., 2012). This suggests that reading is not the driving factor in the formation of spatial–numerical links. Other aspects of the cultural milieu may lead to the development of spatial–numerical links (such as seeing adults model directional counting (Opfer et al., 2010), or utilize gesture in a culturally consistent fashion) which subsequently may be modulated by reading fluency.

### Attentional shifts account

The attentional shift account explains OM as the result of shifts of spatial attention along the MNL that lead participants to prefer outcomes in the “direction” of the arithmetic operation (Knops et al., 2009a). This account predicts that children exhibit a response distribution similar to the pattern observed in adults and a relationship between attentional indices and the strength and/or presence of OM. Children showed an inverse OM bias under a Bayesian approach which is not in line with the predictions of the attentional shift hypothesis. The OM bias was correlated with the reorienting effect. With decreasing reorientation effect the tendency to exhibit a regular OM bias increased across children. Reorientation captures the ability to switch attention from invalidly cued locations to the uncued location at which the actual stimulus appears (Carrasco, 2011). Children who are more proficient in this process tend to show a more adult-like OM bias.

On the neural level in adults reorienting has been associated with joint activation in two distinct but intertwined cortical systems (Corbetta et al., 2008), the ventral attention system (VAS) and the dorsal attention system (DAS). The VAS encompasses right inferior parietal regions around the temporo-parietal junction and ventral frontal cortex including parts of middle frontal gyrus, inferior frontal gyrus, Insula, and frontal operculum (Corbetta et al., 2008). The DAS comprises bilateral areas in the intraparietal sulcus, superior parietal cortex, and frontal eye fields. The DAS is associated with goal-directed orienting of attention that biases the processing of relevant stimuli. In contrast, the VAS is activated by salient but unexpected stimuli and has been proposed to be suppressed during periods of focused attention to prevent reorienting to distracting events. The VAS likely receives filtering information about whether or not an unexpected stimulus is salient from prefrontal cortex (Shulman et al., 2003). Activity in the VAS is modulated at the transition point between two tasks or two stimuli (Dosenbach et al., 2006). Against this background, the size of the reorienting effect might be interpreted as an index for the integrity of the described attentional systems, and/or the extent to which the attentional system is connected to executive control functions (situated in prefrontal cortex). Both reorienting and executive control have been shown to be functional yet immature in 6- to 7-year olds (Konrad et al., 2005; Wetzel et al., 2006, 2009; Carp et al., 2012). Thus children who show a smaller reorientation effect might be more mature in developing situational control over distracting stimuli, and therefore more likely to exhibit an adult-like OM. Together with a functional attentional selection system (DAS) – as evidenced by the significant benefit from valid cues – this implies a key role for a functional and mature attentional system for the OM to arise in the context of non-symbolic calculation tasks. Surprisingly, OM propensity did not correlate with orienting (*r* = 0.14) as predicted by the attentional shift hypothesis. We can only speculate about the reasons for this finding. A regular OM effect may rely on automatic and reliable number-space associations and a full-fledged attentional system. Under this assumption we would speculate to observe increasing OM bias with increasing age. Second, we may hypothesize that correlation between OM and attention increases with age. For the moment these considerations are speculative and need to be addressed in further studies. In this respect the present study presents the first pieces of evidence for the developmental trajectory of the OM effect and its relation to other cognitive domains such as reading and attention. The observed pattern of results is not fully compatible with the attentional shift hypothesis. Nevertheless, the observed response pattern in combination with the significant correlation between reorienting and OM make this hypothesis a promising theoretical approach to delineate the developmental trajectory of the OM effect.

In sum, in the present study we tested several existing hypotheses about the origin of the OM effect by administering a non-symbolic calculation task to children aged between 6 and 7 years. Most crucially, using a Bayesian framework we observed a significant inverse OM effect in children. Testing a sample of college students with the same paradigm revealed a significant regular OM. The children’s results are hard to reconcile with the proposed theoretical accounts for the OM, namely the attentional shift hypothesis, the compression hypothesis, and the heuristic account. The propensity to show a regular OM effect correlated with reorienting scores in an adapted Posner paradigm, linking the OM to the attentional system. We believe that the current child-friendly paradigm offers a promising avenue to further explore the development of spatial–numerical links, and that these findings lead to novel predictions based on the relationship between distinct attention systems, space, and number in both children and adults.

## Conflict of Interest Statement

The authors declare that the research was conducted in the absence of any commercial or financial relationships that could be construed as a potential conflict of interest.
